# Material deprivation and health: a longitudinal study

**DOI:** 10.1186/s12889-016-3327-z

**Published:** 2016-08-08

**Authors:** Anne Grete Tøge, Ruth Bell

**Affiliations:** 1 Department of Social Work, Child Welfare and Social Policy, Faculty of Social Sciences, Oslo and Akershus University College of Applied Sciences, P.O. box 4, St. Olavs plass, NO-0130 Oslo, Norway; 2 Department of Epidemiology & Public Health, Faculty of Population Health Sciences, University College London, 1-19 Torrington Place, London, WC1E 7HB Great Britain UK

**Keywords:** Health, Longstanding illness (LSI), Limiting longstanding illness (LLSI), Social exclusion, Fixed effects

## Abstract

**Background:**

Does material deprivation affect the consequences of ill health? Answering this question requires that we move beyond the effects of income. Longitudinal data on material deprivation, longstanding illness and limiting longstanding illness enables investigations of the effects of material deprivation on risk of limiting longstanding illness. This study investigates whether a shift from affording to not affording a car predicts the probability of limiting longstanding ill (LLSI).

**Methods:**

The 2008–2011 longitudinal panel of Statistics on Income, Social Inclusion and Living Conditions (EU-SILC) is utilised. Longitudinal fixed effects logit models are applied, using LLSI as dependent variable. Transition from affording a car to not affording a car is used as a proxy for material deprivation. All models are controlled for whether the person becomes longstanding ill (LSI) as well as other time-variant covariates that could affect the results.

**Results:**

The analysis shows a statistically significant increased odds ratio of LLSI when individuals no longer can afford a car, after controlling for confounders and LSI in the previous year (1.129, CI = 1.022–1.248). However, when restricting the sample to observations where respondents report longstanding illness the results are no longer significant (1.032, CI = 0.910–1.171).

**Conclusion:**

The results indicate an individual level effect of material deprivation on LLSI, suggesting that material resources can affect the consequences of ill health.

## Background

Numerous studies have observed a close link between poverty and ill health [1–4], and the risk of getting a longstanding illness increases over the life course [5]. However, the extent to which illness leads to limitations in everyday life varies according to several factors, including type, stage and intensity of the health problem, personal perception of the situation, and available economic resources. Using the 2008–2011 panel of EU-SILC, this study tests the hypothesis that material deprivation among the longstanding ill leads to the experience of limitations in their activities.

### Measuring poverty

Does material deprivation affect the consequences of ill health? Answering this question requires that we move beyond the effects of income, because living-standards, wealth/savings and access to goods outside the market also affect access to material resources, and evaluate the effects of material deprivation [6–10]. Poverty is often defined as two main aspects, monetary, i.e., the financial situation and non-monetary, i.e., material deprivation, albeit these two aspects may be difficult to distinguish [11, 12]. Townsend [6] defined poverty as

*… lack the resources to obtain the type of diet, participate in the activities and have the living conditions and the amenities which are customary, or at least widely encouraged or approved in the societies to which they belong. Their resources are so seriously below those commanded by the average family that they are in effect excluded from the ordinary living patterns, customs, and activities.*

This is one of the most influential definitions of poverty and has directly influenced the definition set by the EU Council of ministers in 1985 [11, 12] and the indicators set by the EU Member States and the European Commission to monitor the development in poverty [11, 13]. The items constituting these indicators can be grouped into three areas, i.e., self-reported economic strain; enforced lack of durables (items the household wants but cannot afford) and housing quality. While there is no clear consensus regarding the definition of material deprivation [8] - the nature of deprivation is multi-dimensional, where different dimensions (e.g., education, income and household types) interact [7, 14–16] - one can claim that material deprivation is closely connected to Townsend’s [6] definition of poverty.

Investigating how people change as they become materially deprived or move into a materially deprived area provides an estimate closer to the true causal effects. However, peoples’ self-reported indicators tend to be highly correlated over time, partly due to underlying causes such as mood on the day of interview. In order to avoid such endogeneity in longitudinal analysis, either the indicator of material deprivation or the outcome should be fairly objective measures [17]. This means that an investigation of individual changes in self-reported limitations should apply an objective measure of material deprivation, i.e., none of the measures of self-reported strain. Among the “objective” measures of households’ dwelling facilities and durable, not all are good proxies of current change in material deprivation. Over a short time perspective, the material situation can change without changing the quality of the households’ dwelling. The same can be argued regarding the affordability of a colour TV, a telephone, a washing machine or a computer. After the initial purchase, one does not lose such items by experiencing increased financial difficulties, because the costs of maintaining and using these goods are low. The situation regarding car ownership is different, because the costs of keeping a car and using it are high, which means that ownership and affordability of a car is highly sensitive to financial difficulties [18]. In this study we therefore use a change from affording to not affording a car as a proxy for moving into material deprivation.

While material resources are often assumed to contribute to access to goods that help improve and maintain health, the effect of relative lack of material resources can contribute to psychosocial pathways in health. This is the notion that relative lack of material resources has psychological effects, such as sense of shame, economic worries, and anxiety about being unable to afford the customary living standards of everyday life, that trigger stress and consequent pathways to poor health [19–21]. Changes in the distribution of material resources may therefore affect population health, particularly among those in the lower end of the distribution [4, 9, 10, 20, 22].

In addition to this cross-sectional understanding of outcomes as relative to other respondents/groups, it is also possible to construct a longitudinal understanding of relative, i.e., relative to the previous situation for the same individual. However, few studies have investigated such relationships longitudinally. A study using 1958 British Birth Cohort data found that risk of limiting illness at age 33 is associated with adult socioeconomic disadvantage at age 23, as well as factors including adolescent socio-emotional status, socioeconomic disadvantage in childhood, injury, and poor physical development [23]. Material deprivation indicates a long period of poverty and deprivation as it is much more strongly associated with health than income and other indicators of the socioeconomic status [9, 10].

The aim of this paper is to investigate whether material deprivation contributes to limitations among the longstanding ill. We investigate this question by estimating the individual change in risk of a longstanding illness becoming limiting if people can no longer afford a car.

**Fig. 1:**
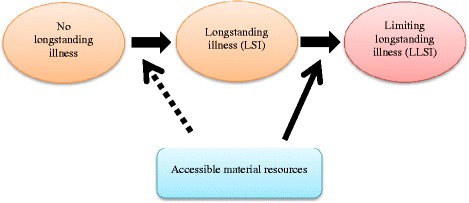
Causal diagram

Figure 1 illustrates the assumed relationship. Processes leading to both longstanding illness (LSI) and limitations in longstanding ill are affected by social determinants, i.e., the economic and social conditions that influence individual health [24, 25], which again affects the risk of limiting longstanding illness (LLSI) [26].

## Methods

This article is based on the 2008–2011 longitudinal panel of Statistics on Income, Social Inclusion and Living Conditions (EU-SILC). EU-SILC is a four-year rotational panel, where 25 per cent of the sample is recruited and 25 per cent is dropped each year. This study utilises the balanced part of the panel, i.e., the respondents that where recruited in 2008 and followed up the next three years. This sample contains 312,556 observations among 78,139 respondents from 27 European countries.^1^ All variables are collected through annual harmonised target variables [27].

### Variables

The aim of this paper is to investigate how individuals’ risk for LLSI changes as their economic situation changes. All variables are therefore time-variant.

#### Dependent variable

The dependent variable is *limiting longstanding illness* (LLSI). This variable is obtained from an item directed to all persons who report that they have a chronic/longstanding illness or condition: “Does this illness or disability (Do any of these illnesses or disabilities) limit your activities in any way?” Yes is coded 1 and no is coded 0. Some studies apply this item as a proxy of severe health impairments or chronic illness [23, 28, 29]. The intention of the item is to measure whether people are hampered in their usual activities by any ongoing physical or mental health problem, illness or disability [27]. Although the item is not meant to measure limitations due to financial, cultural or other none health-related causes, the perception of limitations is highly affected by peoples’ social and economic resources and the societies’ effectiveness at social inclusion. Labour market regulations and social policy affect both employers’ ability to dismiss employees with chronic health conditions, and access to in kind services and financial benefits for individuals with chronic health problems. As such, LLSI is rather a measure of the consequences of ill health than illness itself. Limitations in activities imply restricted possibility to work and participate in social activities, restrictions that could lead to social isolation [30], and affect mental wellbeing [31].

#### Explanatory variable

The main explanatory variable is reduced access to material resources, proxied by not affording a car. Whether people can afford a car is shown to have a clear income gradient [32]. Information on affordability of a car is obtained from the item “Do you have a car?” The values are “yes”, “no, cannot afford one” and “no, other reason”. The variable is given the value 0 “yes” or “no, other reason”, and 1 for “no cannot afford one”.

#### Covariates

Whether the respondent has a *longstanding illness* (LSI) is obtained from the item “Do you have any longstanding illness, disability or infirmity. By longstanding I mean anything that has troubled you over a period of time or that is likely to affect you over a period of time?” The value “yes” is coded 1, while “no” is coded 0.

As change in household structure may affect the affordability of a car, all models control for *partnership* status (coded 1 if the person is married or cohabiting, 0 if not) and number of dependent *children* in the household. *Age* is included to control for the effect of aging on LLSI and *age squared* to adjust for the nonlinear shape of this effect.

### Statistical analysis

Whether transition into material deprivation is associated with increased probability of LLSI is investigated using longitudinal fixed effects logit models, i.e., longitudinal models holding time-invariant factors constant. In this study the respondents’ change in LLSI is a function of change in their affordability of a car. The basic model is:

1 $$ \log \left(\frac{p_{it}}{1-{p}_{it}}\right)={\mu}_i + \beta {\boldsymbol{x}}_{it} + \gamma {\boldsymbol{z}}_i + {\alpha}_i\kern0.5em \mathrm{f}\mathrm{o}\mathrm{r}\ \mathrm{t} = 1, \dots, \mathrm{T}\ \mathrm{and}\ \mathrm{i} = 1, \dots,\ \mathrm{N} $$

where p_it_ is the probability of having LLSI, μ_t_ is a time-varying intercept, **x**_it_ is the time-varying explanatory variable and confounders, **z**_i_ is a vector of time-invariant predictors, and **α**_i_ denotes the combined effect of all unobserved time invariant variables [33]. Because the fixed effects model holds time-invariant factors constant and follows individuals who live in the same country for all of the observed years, cross-national differences and so called “contextual effects” will not affect the estimates unless these factors change over time [34]. Only *changes* in the dependent variable contribute to the estimates [33].

All statistical models are reported with and without control for time-varying covariates, i.e., *partnership*; number of dependent *children*; *age* and *age squared*. As the consequences of material deprivation may take time to manifest, final analyses apply control for a lagged transition, i.e., a transition in the previous year, into not affording a car. “Basic fixed effects models work under the assumption of strict exogeneity, which prohibits some types of feedback from past outcomes to current covariates and current outcome to future covariates” [35]. This possibility of reverse causation is tested by estimating individual changes in the abilities to afford a car as a function of previous changes in LSI and LLSI.

All statistical models are estimated in Stata 14, using the xtlogit command with the fixed effects model option (“, fe”) [33]. The main models are replicated as generalised linear mixed effect models (GLMM), which do not control for time-invariant factors at the individual and country level (reported in Table 7 in Appendix).

## Results

### Descriptive statistics

Table 1 shows summary statistics. Among all observations, 32.6 % come from individuals reporting longstanding illness, while 25.9 % come from individuals reporting their longstanding illness to be *limiting*. During the observational window, 9.6 % of the observations are among individuals who cannot afford a car. The mean age among the respondents at the time of observation is 49.4 years, the mean number of dependent children is 0.93, and 65.6 % are married or cohabiting.

**Table 1:** Summary statistics. *N* = 312,556

Variables	% (n)	N		
Dependent variable:				
Limiting longstanding illness (LLSI)	25.9 (80,709)	312,076		
Explanatory variable:				
Cannot afford a car	9.6 (29,805)	312,076		
Covariates:				
Longstanding illness (LSI)	32.6 (101,833)	312,076		
Partnership	65.6 (204,761)	312,076		
	Mean (SD)	N	Min	Max
Age	49.4 (17.5)	312,076	16	83
Age squared	2744 (1752)	312,076	0.265	6.889
Children	0.93 (1.31)	312,076	0.000	15.000

Table 2 shows yearly shares of respondents who cannot afford a car in the entire sample, in respondents with LSI and in respondents with LLSI. The share of respondents that cannot afford a car has decreased in all groups.

**Table 2:** Cannot afford a car by year in all respondents, respondents reporting LSI and respondents reporting LLI

		Cannot afford a car			
		2008	2009	2010	2011
All	%	10.0	9.7	9.4	9.1
	n	78,019	78,019	78,019	78,019
LSI	%	12.1	11.1	10.7	10.3
	n^a^	24,382	24,936	25,800	26,715
LLI	%	13.2	12.5	12.5	11.9
	n^a^	19,056	19,699	20,254	21,700

^a^Increasing because the prevalence of illness increases as people get older

### Test for reverse causality

The thrust of the paper is that material deprivation is associated with increased risk of LLSI, however it could be that the relationship is the other way around. If LLSI causes material deprivation rather than the other way around, than the temporal order should be higher probability of not affording a car *after* getting LLSI. Table 3 reports odds ratios (CI) of not affording a car as a function of changes in LLSI in the previous year. LLSI is significantly negatively correlated with not affording a car (Model 1a), i.e., becoming limiting longstanding ill in the previous year is associated with increased affordability of a car. The estimate is still significant when controlled for confounders (Model 1b), and becomes even stronger when controlling for changes in LSI at *t-1* (Models 2a and 2b). A control for LSI at *t-1* implies holding changes in LSI at previous constant, i.e., controlling whether the respondent moved into LSI in the year before the household could no longer afford a car, which could be an important confounding factor. Table 3 reveals that controlling for confounders - including LSI - a shift into LLSI one year is associated with decreased probability of not affording a car the next year. We control for transition into LSI in years before *t-1* because EU-SILC is a four-year panel. An inclusion of LSI at *t-2* instead of *t-1* implies that the model only investigates the change in LLSI from 2010 to 2011. Nevertheless, when we control for this change, the results are no longer significant. Whether this is due to the short panel or the appropriate inclusion of control for “LSI at t-2” is not possible to determine.

**Table 3:** Cannot afford a car, as a function of previous change in LSI and LLSI. Longitudinal fixed effects logit models

	1a	1b	2a	2b
Variables	Cannot afford a car	Cannot afford a car	Cannot afford a car	Cannot afford a car
	Odds ratio (95 % CI)	Odds ratio (95 % CI)	Odds ratio (95 % CI)	Odds ratio (95 % CI)
Limiting longstanding illness (LLSI), (t-1)	0.897**	0.912*	0.868**	0.881**
	(0.812–0.990)	(0.825–1.008)	(0.777–0.969)	(0.788–0.984)
Longstanding illness (LSI), (t-1)			1.078	1.085
			(0.965–1.205)	(0.971–1.213)
Covariates:				
Partnership, children, age, age squared	No	Yes	NO	Yes
	NO	NO	Yes	Yes
Number of observations	19,767	19,767	19,767	19,767
Number of respondents	6589	6589	6589	6589

* = *p* < 0.10, ** = *p* < 0.05 & *** = *p* < 0.01

### Results from multivariate analyses

Table 4 reports the individual change in LLSI as a function of moving into a state where the respondent can no longer afford a car. Model 3a shows a minor, but not statistically significant, increased odds ratio of LLSI (1.045, CI = 0.959–1.139) when individuals no longer can afford a car. This increased odds ratio is still not significant when controlling for confounders (1.064, CI = 0.976–1.160, Model 3b). Models 4a and 4b include a one year period lag of the LSI, and show a significant increased odds ratio of LLSI when individuals no longer can afford a car in both the uncontrolled (1.111, CI = 1.006–1.227) and controlled model (1.129, CI = 1.022–1.248, full model is shown in Table 6 in Appendix).

**Table 4:** Change in LLSI as a function of not affording a car and changes in LSI. Longitudinal fixed effects logit models

	3a	3b	4a	4b
	LLSI	LLSI	LLSI	LLSI
Variables	Odds ratio (95 % CI)	Odds ratio (95 % CI)	Odds ratio (95 % CI)	Odds ratio (95 % CI)
Cannot afford a car	1.045	1.064	1.111**	1.129**
	(0.959–1.139)	(0.976–1.160)	(1.006–1.227)	(1.022–1.248)
Covariates:				
Partnership, children, age, age squared	No	Yes	No	Yes
Longstanding illness (LSI) at t	Yes	Yes	No	No
Longstanding illness (LSI) at t-1	No	No	Yes	Yes
Number of observations	93,744	93,744	55,110	55,110
Number of respondents	23,436	23,436	18,370	18,370

* = *p* < 0.10, ** = *p* < 0.05 & *** = *p* < 0.01

### Sensitivity analysis

Table 5 presents models where LSI is held constant by restricting the sample to observations where respondents report longstanding illness (LSI). Model 5a shows a small, but not statistically significant, increased odds ratio of LLSI (1.008, CI = 0.889–1.143) when individuals no longer can afford a car. This increased odds ratio is a bit steeper when controlling for confounders (1.032, CI = 0.910–1.171, Model 5b), but still not statistically significant. The direction of the results in Table 5 support the findings in Table 4, however, the correlations are weaker and not statistically significant.

**Table 5:** Change in LLSI as a function of not affording a car. Longitudinal fixed effects logit models. Sample restricted to individual reporting LSI

	5a	5b
	LLSI	LLSI
Variables	Odds ratio (95 % CI)	Odds ratio (95 % CI)
	LLSI	LLSI
Cannot afford a car	1.008	1.032
	(0.889–1.143)	(0.910–1.171)
Covariates:		
Partnership, children, age, age squared	No	Yes
Number of observations	31,257	31,257
Number of respondents	9559	9559

* = *p* < 0.10, ** = *p* < 0.05 & *** = *p* < 0.01

## Discussion

The main finding is that moving into material deprivation - measured as no longer affording a car – contributes to a restriction in everyday life among people with chronic illness. The study suggests that material deprivation restricts people from doing what they would have normally done, e.g., go to work and engage in physical and social activities. As such, this study contribute to the discussion of the causal effects of poverty on health related social exclusion. Retired and older people may be at particular risk of social isolation, and three life events are critical: retirement and losing connection with colleagues; falling ill and becoming less mobile; a spouse dying or going into care [36, 37]. Enabling particularly older people with chronic illness to participate in society might therefore mitigate both social and economic costs of ill health [38].

Albeit the current study investigates the effect of an “absolute” change from affording to not affording a car, a relative dimension is embedded in the longitudinal design; the change is relative to previous situation for the same individual. Affording a car in year *t* will not contribute to the estimates if the person also could afford a car (or not needed it) in the previous year [*t-1*], it is only a change from affording to not affording a car that can explain change in LLSI. The strength of this design is that it contributes to the understanding of how life events and changing living conditions affect health and the consequences of ill health. Several studies have suggested that the composition of the local population has an effect beyond individual characteristics [34, 39, 40]. This study adds to this knowledge by substantiating the causal pathway from material deprivation to health related social exclusion.

Nevertheless, when restricting the analysis to the longstanding ill and controlling for household structure and age, cannot afford a car is no longer a significant predictor of LLSI. This could be explained by underlying causes or social determinants that affect both material deprivation and experienced limitations [24, 25, 41]. This study could imply that such underlying factors, e.g., peoples’ ability to remain their social network and relationships, could be an important factor in explaining the effect of material deprivation on LLSI.

Contextual factors e.g., accessibility and affordability of public transport, support from family, friends, local community, social capital in area of residence (extent of connectivity), and national policies to support people with LLSI) will also affect whether people need a car [42]. Investigations of contextual effects suggest that the prevalence of material deprivation, educational level and the share of lone parent households predict self-rated health at the individual level [34, 39]. Even within Norway, which is believed to be a very egalitarian country, Elstad [40] found that the broader socio-economic context affected regional mortality levels. All these studies suggest that the negative health effects of poverty diffuse into the environment in line with the prevalence of the phenomenon. Studies of contextual variation also suggest that this negative effect can be counteracted by social policy. A multilevel study of health related social exclusion among disadvantaged groups in Europe found lower risk of non-participation in social networks in countries with more generous provision of social security [43]. Labour market inclusion and reduced poverty among the unemployed could be particularly important. A repeated cross-sectional study found higher prevalence of poverty among people with LLSI in the United Kingdom than in the more regulated Sweden and Denmark, and greater increase in poverty among people with LLSI in Sweden, that has cut back social benefits, than in Denmark and United Kingdom. The effect of moving into material deprivation may therefore be affected by several contextual factors, including social cohesion, the prevalence of disadvantages in the local area and labour market regulations and quality and generosity of social policies. The current study applies fixed effects models, separates between the reasons for not owning as “cannot afford” and “other reasons”, and uses ‘cannot afford a car’ as a proxy for moving into material deprivation, therefore the interpretation of the estimates allows for an interpretation closer to the “real” causal effects of material deprivation. A decline in material living standards leads to elevated risk of limitations among people with chronic illness. As such, the results indicate that the current social policies cannot fully protect individuals with longstanding illness from the consequences of a decline in material living standards.

Nevertheless, “cannot afford a car” as a proxy of material deprivation cannot separate between the effects of relative deprivation and absolute poverty. No longer affording a car could imply that the family no longer prioritise having a car, in order to keep up other standards, or it could imply that their economic resources are so scarce that they could not afford a car even if they reduced other standards. As the affordability of a car only indicates the affordability of a car and not a latent construct of deprivation and living standards, there is a risk of measuring absolute material hardship rather than more complex and multidimensional aspects of poverty.

Attrition from panel studies are always problematic and can cause biased estimates [44]. EU-SILC has a rotational design, where new respondents are recruited each year and followed for up to four years. The raw panel is therefore naturally unbalanced. In the absence of a variable that assigns whether a respondent is missing because of attrition or because the last observation was the scheduled last, it is difficult to determine the impact of attrition in this study. Nevertheless, each initial sample is based on a nationally representative probability sample, where the reference population is all private households and all persons aged 16 and over. Investigations of attrition in the European Community Household Panel (ECHP), which was the precursor to EU-SILC found that moving and change of interviewer were the most prominent predictors of attrition [45].

Albeit longitudinal analysis can establish a temporal correlation, the challenge of detecting the causal direction of the relationship remains [35]. There is limited evidence for a reverse causality, i.e., LLSI causing a move from affording to not affording a car. The evidence for a causal effect of material deprivation on LLSI would be strengthened if the study could establish the temporal order; if a person gets ill health [*t*], remains ill and cannot afford a car [*t + 1*], and then becomes *limiting* longstanding ill [*t + 2*]. However, the EU-SILC panel is too short to provide statistical power for such an investigation.

## Conclusion

The results indicate an individual level effect of material deprivation on LLSI, and thereby illustrates the importance of material resources in keeping people with LSI free of limitations for as long as possible. This study does not provide any evidence for possible effective prevention, however, it suggests that further research should investigate effects of policies and schemes aimed at reducing the negative health effects of poverty and material deprivation.

## Abbreviations

CI, confidence intervals; EU-SILC, EU Statistics on Income, Social Inclusion and Living Conditions; LLSI, limiting longstanding illness; LSI, longstanding illness

## Appendix

**Table 6:** Change in LLSI as a function of not affording a car and changes in LSI. Longitudinal fixed effects logit models

	4b
	LLSI
Variables	Odds ratio (95 % CI)
Cannot afford a car	1.129**
	(1.022–1.248)
Covariates:	
Longstanding illness (LSI) at t-1	0.652***
	(0.622–0.683)
Partnership	1.004
	(0.853–1.182)
Children	0.982
	(0.952–1.014)
Age	0.953
	(0.891–1.019)
Age squared	1.002***
	(1.001–1.003)
Number of observations	55,110
Number of respondents	18,370

* = *p* < 0.10, ** = *p* < 0.05 & *** = *p* < 0.01

**Table 7:** Change in LLSI as a function of not affording a car and changes in LSI. Generalised mixed effects models

	3a	3b	4a	4b
	LLSI	LLSI	LLSI	LLSI
Variables	Odds ratio (95 % CI)	Odds ratio (95 % CI)	Odds ratio (95 % CI)	Odds ratio (95 % CI)
Transition into cannot afford a car^a^	1.372***	1.303***	1.428***	1.377***
	(1.297–1.452)	(1.228–1.383)	(1.356–1.504)	(1.304–1.455)
Covariates:				
Partnership, children, age, age squared	No	Yes	No	Yes
Longstanding illness (LSI) at t	Yes	Yes	No	No
Longstanding illness (LSI) at t-1	No	No	Yes	Yes
Random-effects				
Level 3: Country	0.180	0.207	0.116	0.144
	(0.083–0.276)	(0.095–0.319)	(0.053–0.179)	(0.066–0.221)
Level 2: Respondent	1.300	1.306	0.928	0.995
	(1.266–1.334)	(1.270–1.342)	(0.900–0.962)	(0.962–1.028)
Number of observations	312,076	312,076	234,057	234,057
Number of respondents	78,019	78,019	78,019	78,019
Number of countries	27	27	27	27

* = *p* < 0.10, ** = *p* < 0.05 & *** = *p* < 0.01

^a^The variable only includes the *transition into not affording a car*, not *transition from not affording a car* to *affording a car*. The variable is coded 1 the first year [*t*] a transition from “*affording a car*” or “do not have a car because of other reasons” [*t-1*] to “no cannot afford one” [*t*] is observed. All observations after the transition are coded 1 in order to avoid transition back to affording a car contributing to the estimates. All other observations are coded 0

## Footnotes

1 Romania, Hungary, Sweden, Bulgaria, Finland, Austria, Italy, Latvia, Portugal, Cyprus, Czech Republic, Norway, Malta, Slovenia, Netherlands, Slovakia, France, Lithuania, Greece, Luxembourg, Poland, Spain, Belgium, Denmark, Estonia, Iceland and United Kingdom. Croatia joined in 2009, and are therefore not included.
